# A systematic review and meta-analysis of the use of resuscitative endovascular balloon occlusion of the aorta in the management of major exsanguination

**DOI:** 10.1007/s00068-018-0959-y

**Published:** 2018-05-21

**Authors:** B. L. S. Borger van der Burg, Thijs T. C. F. van Dongen, J. J. Morrison, P. P. A. Hedeman Joosten, J. J. DuBose, T. M. Hörer, R. Hoencamp

**Affiliations:** 1 Department of Surgery, Alrijne Hospital, Simon Smitweg 1, 2353 GA Leiderdorp, The Netherlands; 2 Defense Healthcare Organization, Ministry of Defense, Utrecht, The Netherlands; 3 R. Adam Cowley Shock Trauma Center, University of Maryland Medical System, Baltimore, USA; 4 Division of Vascular Surgery, David Grant Medical Center, Travis AFB, California, USA; 5 Department of Cardiothoracic and Vascular Surgery, Örebro University Hospital, Örebro University, Örebro, Sweden; 6 Division of Surgery, Leiden University Medical Centre, Leiden, The Netherlands

**Keywords:** Trauma, Shock, Endovascular, Aortic balloon occlusion, REBOA

## Abstract

**Background:**

Circulatory collapse is a leading cause of mortality among traumatic major exsanguination and in ruptured aortic aneurysm patients. Approximately 40% of patients die before hemorrhage control is achieved. Resuscitative endovascular balloon occlusion of the aorta (REBOA) is an adjunct designed to sustain the circulation until definitive surgical or endovascular repair. A systematic review was conducted for the current clinical use of REBOA in patients with hemodynamic instability and to discuss its potential role in improving prehospital and in-hospital outcome.

**Methods:**

Systematic review and meta-analysis (1900–2017) using MEDLINE, Cochrane, EMBASE, Web of Science and Central and Emcare using the keywords “aortic balloon occlusion”, “aortic balloon tamponade”, “REBOA”, and “Resuscitative Endovascular Balloon Occlusion” in combination with hemorrhage control, hemorrhage, resuscitation, shock, ruptured abdominal or thoracic aorta, endovascular repair, and open repair. Original published studies on human subjects were considered.

**Results:**

A total of 490 studies were identified; 89 met criteria for inclusion. Of the 1436 patients, overall reported mortality was 49.2% (613/1246) with significant differences (*p* < 0.001) between clinical indications. Hemodynamic shock was evident in 79.3%, values between clinical indications showed significant difference (*p* < 0.001). REBOA was favored as treatment in trauma patients in terms of mortality. Pooled analysis demonstrated an increase in mean systolic pressure by almost 50 mmHg following REBOA use.

**Conclusion:**

REBOA has been used in trauma patients and ruptured aortic aneurysm patients with improvement of hemodynamic parameters and outcomes for several decades. Formal, prospective study is warranted to clarify the role of this adjunct in all hemodynamic unstable patients.

**Electronic supplementary material:**

The online version of this article (10.1007/s00068-018-0959-y) contains supplementary material, which is available to authorized users.

## Introduction

Controlling catastrophic bleeding is the major life saving skill in trauma and vascular surgery. The revival of the tourniquet for management of extremity bleeding and massive transfusion protocols found their basis on the battlefields of Iraq and Afghanistan [1], however, difficult anatomical locations such as the neck, truncal and ilio-junctional regions continue to represent challenges for prompt bleeding control. Truncal and junctional hemorrhage are often described within the trauma literature as non-survivable injuries contributing for almost 90% of catastrophic hemorrhage fatalities in the prehospital phase, in contrast to a 10% fatality rate in extremity injuries [2–4]. Bleeding from other non-compressible sites not related to trauma, such as the gastrointestinal tract, the post-partum uterus, a ruptured abdominal aortic aneurysm (rAAA), and traumatic disruption of thoracic, abdominal, or pelvic viscera can also represent similar challenges for prompt direct bleeding control and have traditionally been characterized as potentially non-survivable sources of hemorrhage. rAAA represents a classic source of non-traumatic major hemorrhage and has been associated with reported in-hospital mortality rates occurring in 30% of patients treated with EVAR and 42% of the patients undergoing open repair [5]. According to the Dutch Surgical Aneurysm Audit (DSAA) the number of patients that were treated in the Netherlands for a ruptured aortic aneurysm in 2013 was 293, with an in-hospital mortality of 35.3% [6]. It has also been demonstrated that approximately 25% of rAAAs undergo complete circulatory collapse before or during the procedure. The natural history of this uncontrolled hemorrhage has been shown to be cardiovascular collapse with consequent cerebral and myocardial hypoperfusion, ultimately leading to death [7, 8].

Endovascular balloon occlusion of the aorta is a technique where a compliant balloon is advanced into the aorta and then inflated, thereby obstructing flow into the distal circulation. This has the effect of increasing cardiac afterload and proximal aortic pressure, resulting in an increase in myocardial and cerebral perfusion [9]. This technique was first described by Hughes in 1954 when an intra-aortic balloon catheter tamponade was utilized in two moribund Korean War casualties with uncontrolled intra-abdominal hemorrhage [10].

In 1964, Heimbecker reported the first use of an aortic tampon for emergency control of a ruptured abdominal aneurysm [11].

Recent champions of the endovascular resuscitation and trauma management (EVTM) concept have re-introduced the concept of using resuscitative endovascular balloon occlusion of the aorta (REBOA) to modern clinical practice [12]. Two recent systematic analysis by these investigators have helped to consolidate a disparate evidence base [13, 14]. However, a clear mortality benefit has yet to be demonstrated and consequently, the REBOA concept is still not fully embedded as the standard of care in most hospitals.

Furthermore, the pioneering use of this hemorrhage control adjunct in the prehospital phase, despite early promising results [15, 16], has not been widely adopted. Recent terrorist threats in western countries have contributed to an increased awareness of the value for bleeding control in the earliest phases after injury, suggesting that the potential utilization of this REBOA in this setting warrants examination [17]. To date, however, a complete analysis of all international published literature has not been conducted. In particular, there is a need for an examination of focusing on differences between the main indications; trauma and non-trauma related major hemorrhage (including rAAA).

The primary aim of this systematic review was to examine the use of REBOA and the mortality and morbidity associated with REBOA in patients with hemodynamic instability due to major exsanguination from both traumatic and non-traumatic sources. The secondary aim was to provide an evidence-based rationale for optimal utilization of REBOA in the in-hospital phase and, ultimately contribute to the discussion of the potential for this technology to be more aggressively employed as an on-scene adjunct for control of major hemorrhage in both trauma and non-trauma settings.

## Methods

The protocol for objectives, literature search strategies, inclusion and exclusion criteria, outcome measurements, and methods of statistical analysis was prepared a priori, according to the Preferred Reporting Items for Systematic Reviews and of Observational Studies in Epidemiology recommendations for study reporting and is described in this section [18, 19].

### Search strategy and selection criteria

The MEDLINE, Cochrane, EMBASE, Web of Science and Central and Emcare databases were searched for relevant articles published from January 1900 to September 2017, using the Ovid medical search engine. The keywords used in the search were composed of combinations of “aortic balloon occlusion”, “aortic balloon tamponade”, “REBOA”, “Resuscitative Endovascular Balloon Occlusion” in combination with hemorrhage control, hemorrhage, resuscitation, shock, ruptured abdominal, thoracic aorta, endovascular repair, open repair. The search was limited to original studies on human subjects (abstracts), published in English language journals.

### Inclusion and exclusion criteria, data extraction and outcomes of interest

Three reviewers (B.B.B., T.D. and R.H.) independently screened the abstracts for suitability. Publications were excluded where non-ruptured aortic aneurysms were reported or ineligible study types (e.g., letters and reviews) were identified. Following abstract screening, the remaining publications were subjected to a full-text assessment. The full-text assessment consisted of two reviewers independently assessing the publication to determine suitability for inclusion. Level I–V evidence was considered, and particular attention was paid to the reporting of hemodynamic performance, balloon type, balloon deployment technique, complications, and mortality. During the full-text assessment, articles were excluded if little or no clinical data were reported on the subject of balloon occlusion. Where articles undergoing full-text review identified additional relevant studies that had not been previously identified, the additional relevant studies were also reviewed and included if eligible. If disagreement was encountered at any stage of the publication inclusion/exclusion process, the senior reviewer (RH) and a fourth independent reviewer (P.H.J) arbitrated a final decision.

### Quality assessment

Studies were rated for the level of evidence provided according to criteria by the Centre for Evidence Based Medicine in Oxford. The risk of bias was assessed for each study using the Cochrane Collaboration’s tool for assessing the risk of bias. This tool assesses six domains (selection, performance, detection, attrition, reporting, and other) and rates the risk of bias in each as “high”, “low”, or “unclear” [14]. The key characteristics of the eligible studies in terms of study type, clinical setting, aortic zone of occlusion, mortality, and morbidity were extracted as described earlier and summarized in a tabular format. These studies are the best evidence of mortality and morbidity associated with REBOA published up to 2017.

### Statistical analysis

The categorical variables were analyzed by their absolute and relative frequencies in percentages. For the trauma cohort, we performed a meta-analysis to calculate the effect on systolic blood pressure and mortality. Only studies were REBOA was used as a primary means to control blood loss and contained an adequate control group, were used in the meta-analysis. The association between two categorical variables was calculated by applying the Pearson Chi-square test or *I*^2^ test. We used with fixed effect and used *Χ*^2^/*I*^2^ for heterogeneity. The software package SPSS (24.0, IBM Corporation, Armonk, New York), was used for statistical analysis to achieve a combined outcome. In all cases, *p* < 0.05 (two-sided) was considered statistically significant.

## Results

**Fig. 1:**
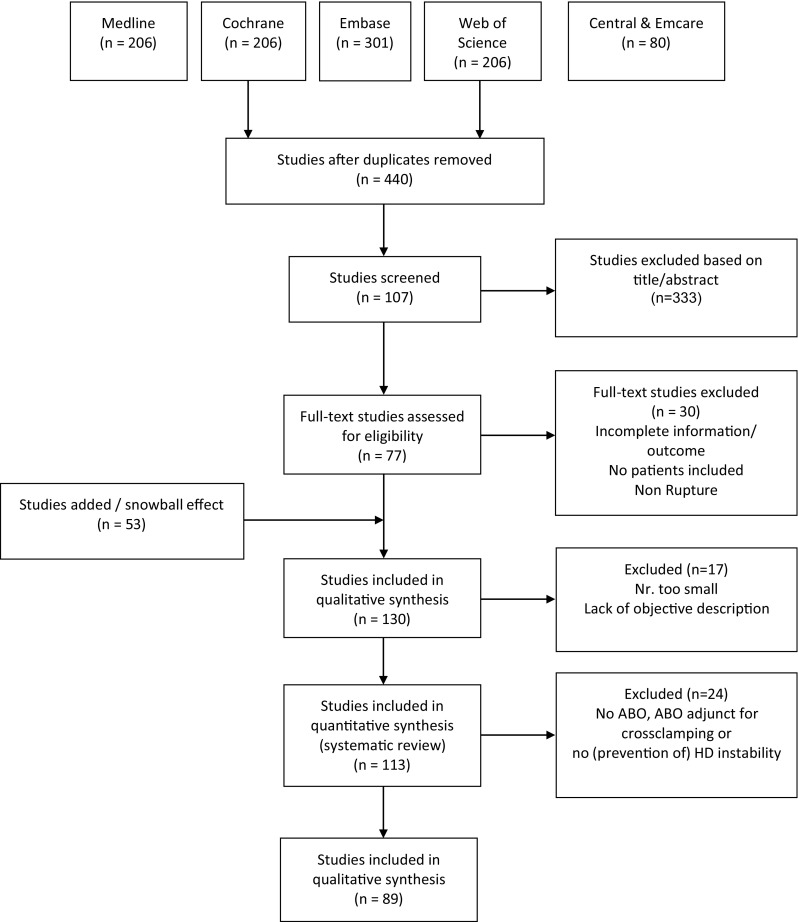
PRISMA flow chart for the systematic review. *n* indicates number, *ABO* aortic balloon occlusion, *HD* hemodynamic

A total of 440 unique studies were identified and underwent abstract screening (Fig. 1), 107 of these studies were deemed appropriate for and underwent full-text review. Of these, 30 studies reported little or no clinical data, leaving 77 studies for inclusion following full-text review. Reference review of these studies identified an additional 53 eligible studies, which were not identified during the key word search. These studies were predominantly older in publication date but were still eligible for inclusion. Therefore, after final quantitative synthesis, 89 articles were selected, including 28 case reports [11, 20–46], 25 case series [10, 47–71], and 36 (retrospective) cohort studies [12, 72–105]. Also 8 reviews were identified but not included in calculations, due to concern for double counting of unique cases [5, 13, 14, 106–110]. Most studies were deemed to be at high risk of bias. In total, 1482 patients treated with REBOA were included in analysis (Table 1).

**Table 1:** Included studies in systematic review

Reference	Year	Study type	*N* Balloon (Tot)	Aortic zone placement	Shock	Mortality (in-hosp)	AO related iatrogenic injury	Risk of bias	OXLE
Case report (28)									
Armour	1978	rAAA	1	I	Y	0	Nil	NA	V
Bell-Thomas	2003	PPH	1	III	Y	0	Nil	NA	V
Cakir	2014	rAAA	1	I	Y	0	Nil	NA	V
D’Hondt	2008	R aneurysma aortobifem graft	1	III	Y	0	Nil	NA	V
Green	2014	Trauma abdpel. hem	1	III	N	0	Nil	NA	V
Harma	2004	PPH	1	III	Y	0	Nil	NA	V
Heimbecker	1964	rAAA	1	III	Y	0	Nil	NA	V
Hesse	1962	rAAA	1	III	Y	1/1	Nil	NA	V
Hill	2010	UGI bleeding	1	I	Y	0	Nil	NA	V
Howard	1972	rAAA	1	III	Y	0	Nil	NA	V
Karkos	2001	UGI bleeding	1	I	Y	0	Nil	NA	V
Lai	2008	rAAA	1	I	Y	0	Nil	NA	V
Lee	2016	GI bleeding	1	I	Y	0	Nil	NA	V
Malina	2005	rAAA	1	I	Y	0	Nil	NA	V
Masamoto	2009	PPH	1	III	N	0	Nil	NA	V
Matsuoka	2001	Trauma abdpel. Hem	1	I	Y	0	Nil	NA	V
Menke	2010	r-Para-anastomotic iliac aneur	1	III	Y	0	Nil	NA	V
Namura	2001	rAAA	1	III	Y	0	Nil	NA	V
Ozgiray	2009	Pelvic bleeding peri-OK	1	III	N	0	Nil	NA	V
Paull	1995	PPH	1	III	N	0	Nil	NA	V
Pettersson	2003	r. pseudo aneurysma aorta asc	1	I	Y	0	Nil	NA	V
Schumacher	2004	rAAA	1	II	Y	0	Nil	NA	V
Shigesato	2015	UGI	1	I	Y	0	Nil	NA	V
Smith	1972	rAAA	1	III	Y	0	Nil	NA	V
Soda	2010	Pelvic bleeding peri-OK	1	III	N	0	Nil	NA	V
Uchida	2014	Trauma abdpel. hem	1	III	Y	0	Nil	NA	V
Wolf	1986	Trauma abdpel. hem	1	III	Y	0	Nil	NA	V
Xiong	2014	Pelvic bleeding peri-OK	1	III	N	0	Nil	NA	V
Total			28		22/28	1/28	0		
Case series (25)									
Arthurs	2006	rAAA	3	I	N	0/3	Nil	High	IV
Brenner	2013	Trauma abdpel. hem	6	Ix4, IIIx2	Y	2/6	Nil	High	IV
Delalieux	2010	rAAA	1 (18)	U	Y	U (7/18)	Nil	High	IV
Greenberg	2000	rAAA	2 (3)	I	Y	0/2	Nil	High	IV
Guo	2009	rAAA	4 (26)	II/III	Y	U (7/26)	Nil	High	IV
Gupta	1989	Trauma abdpel. Hem	21	I	Y	14/21	1× fem a thrombosis	High	IV
Hinchliffe	2001	rAAA	2 (20)	I	Y	U (9/20)	Nil	High	IV
Hughes	1954	Trauma abdpel. hem	2	I	Y	2/2	Nil	High	IV
Irahara	2015	Trauma abdpel. hem	14	I	Y	9/14	Nil	High	IV
Lagana	2006	rAAA	3 (30)	II	Y	U (3/30)	NR	High	IV
Lee	2008	rAAA	3 (52)	U	Y	U (28/52)	Nil		IV
Martinelli	2010	Trauma abdpel. hem	13	III	Y	7/13	1x fem a thrombosis, 1x balloon rupture	High	IV
Matsuda	2003	rAAA	11 (11)	III	Y	3/11	3x bal. rupture, 2x embolic complication	High	IV
Mayer	2009	rAAA	19	I	Y	U	1x embolic complication	High	IV
Ng	1977	rAAA	5	III	Y	3/5	Nil	High	IV
Ogura	2015	Trauma abdpel. hem	35	I	Y	16/35	Nil	High	IV
Ohki	2000	rAAA	9	I	Y	U	Nil	High	IV
Philipsen	2009	rAAA	12	I	Y	1/12	Nil	High	IV
Sensenig	1981	rAAA	3	I/II/I	Y	0/3	Nil	Low	IV
Sovik	2012	PPH	6	III	Y	0/6	1x Aortic injury	High	IV
Taheri	1988	rAAA	2	III	N/Y	0/2	Nil	Low	IV
Wang	2013	Trauma abdpel. hem	5	III	Y	U	Nil	High	IV
Xue-Song	2010	Pelvic bleeding peri-OK	9	III	N	0/9	Nil	High	IV
Yang	2008	Pelvic bleeding peri-OK	12	III	N	0/12	Nil	High	IV
Zhang	2007	Pelvic bleeding peri-OK	5	III	N	0/5	Nil	High	IV
Total			207 (341)		177/207	57/161	10		
Cohort study (36)									
Alsac	2005	rAAA	1 (37)	I	Y	U (14/37)	Nil	High	
Anain	2007	rAAA	12 (40)	I	Y	5/12	Nil	High	IV
Carafiello	2012	rAAA	4 (42)	U	Y	U (13/42)	Nil	High	IV
Coppi	2006	rAAA	4 (124)	II	Y	U (73/142)	Nil	High	IV
Dalainas	2006	rAAA	28	II	5/20	8/28	Nil	High	IV
Djavani G	2011	rAAA	2 (29)	U	Y	1/2	Nil	High	IV
DuBose	2016	Trauma	46 (114)	Ix33, IIx1, IIIx8	21/46	13/46	2x embolism, 1xpseudo aneurysm	High	IV
Gerassimidis	2008	rAAA	2 (41)	U	Y	U (15/41)	Nil	High	IV
Holst	2009	rAAA	23 (90)	I	Y	U (24/90) (BOA corr sign,30d)	2x SMA coverage	High	IV
Hörer	2015	Trauma/Other	11	NA 2x iatr, 2 gyn, 7 trauma	Y	4/11	3 × (2× perf. (iliaca/fem), uncontroled ECMO removal	High	IV
Hörer	2017	Trauma	96	Ix86, IIx3, IIIx3	43/65	54/96	13 (miscellaneous)	High	IV
Ioannidis	2012	rAAA	1 (20)	U	Y	U (10/20)	Nil	High	IV
Karkos	2008	rAAA	2 (41)	U	Y	U (17/41)	Nil	High	IV
Larzon	2005	rAAA	13 (41)	I	Y	U (14/41)	Nil	High	IV
Low	1986	Trauma Abd-pelvic/rAAA//oth	22 (15/5/2)	I	Y	17/22 Overall 13/15 Trauma 1/5 rAAA 2/2 Other	5x perc access failure, 1x cut down failure	High	IV
Luo	2013	Pelvic bleeding peri-OK	45	III	N	0/45	3x fem a thrombosis	High	IV
Matsumara	2017	Trauma	106	Ix99, IIx5, IIIx2	Y	38/106	Nil	Low	III
Mayer	2012	rAAA	62 (268) (EVAR only)	I	Y	U (48/268)	Nil	High	III
Mehta 2005	2005	rAAA	7 (30)	I	Y	U (7/30)	Nil	High	IV
Mehta 2013	2013	rAAA	23 (136)	I	Y	U (32/136)	Nil	High	IV
Moore	2006	rAAA	7 (20) (EVAR only)	II	Y	1/7	Nil	High	IV
Moore	2015	Trauma abdpel. hem	24	Ix19, IIIx5	Y	15/24	Nil	High	IV
Mukherjee	2014	rAAA	3 (47)	I	Y	U (12/55)		High	IV
	2014	rAAA (hybrid group)	8	I	3/8	0/8	Nil	Low	IV
Nedeau	2012	rAAA	11 (74)	U	Y	U (30/74)	Nil	High	IV
Norii	2015	Trauma abdpel. hem	452	U	Y	343/452	Nil	High	III
Ockert	2007	rAAA	2 (58)	U	Y	U (18/58)	Nil	High	IV
Peppelenbosch	2005	rAAA	7 (100)	II	Y	U (37/100)	Nil	High	IV
Raux	2015	rAAA	32 (72)	II/III	Y	22/32	Nil	Low	III
Resch	2003	rAAA	5 (21)	U	Y	U (4/21)	Nil	High	IV
Saito	2014	Blunt trauma	24	I-III	Y	17/24	5× failure of infl 1× iliac injury	Low	IV
Sarac	2011	rAAA	3 (32)	U	Y	0/3	Nil	High	IV
Starnes	2010	rAAA	11 (179)	U	Y	7/11	Nil	High	IV
Takagi	2003	Aortic Arch repair (ruptured only)	8	I	Y	3/8	Nil	High	IV
Tang	2010	Pelvic bleeding peri-OK	120	III	N	0/120	3x fem a embolism, 5x puncture site hematoma	High	III
Veith	2002	rAAA	10 (31)	U	Y	U (3/31)	Nil	High	IV
Veith	2003	rAAA	10 (36)	I	Y	U (26/36)	Nil	High	IV
Total			1247 (2659)		976/1216	555/1057	44		
Grand total (*n* = 89 studies)			1482 (2960) (50.1%)		1175/1482 (79.3%)	613/1246 (49.2%)	54		

*EVAR* endovascular aneurysm repair, *N* number, *in-hosp* in hospital, *AO* “Arbeitsgemeinschaft für Osteosynthesefragen”, *OXLE* Oxford level of evidence, *Art.Ins* location of arterial incision, *tech* technique, *Perc* percutaneous, *Occl* occlusion, *SD* standard deviation, *Δ P mmHg* pressure difference in mmHg, *rAAA* ruptured abdominal aneurysm, *abdpel hem* abdominal/pelvic haemorrhage, *PPH* post partum haemorrhage, *(U)GI* (upper) gastro intestinal, *OR* operation room, *r*. ruptured, *iatr*/*gyn* iatrogenic/gynaecological, *Y* yes, *NA* not applicable, *U* unknown, *nm* not measurable, *Ao* aortal, *Ax* axillary, *B* brachial, *C* carotis, *F* femora, *(I)AOB* (intra) aortic occlusion balloon, *bal* balloon, *cath* catheter, *pts* patients, *fem a*. femoral artery, *def* deflation, *pt(s)* patient(s)

### Indications, hemodynamic instability and mortality

REBOA has been used for the management of hemorrhage grouped in three major clinical indications: (1) traumatic abdominopelvic hemorrhage (18 studies), (2) hemorrhage arising from rAAA (50 studies) and, (3) miscellaneous causes such as post-partum, gastro-intestinal bleeding or exsanguination during pelvic surgery (21 studies). The REBOA concept was used in all studies as a hemorrhage control and resuscitation adjunct for prompt hemorrhage control.

**Fig. 2:**
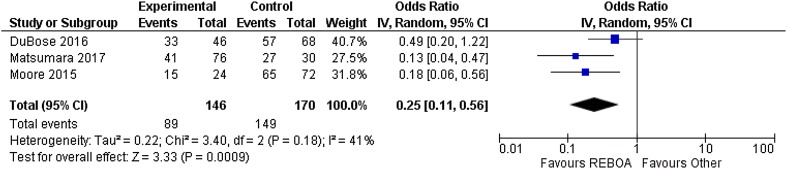
Meta-analysis of mortality after use of REBOA in trauma. *REBOA* indicates resuscitative endovascular balloon occlusion of the aorta, *IV* inverse variance, *Random* random effect, *CI* confidence interval, *df* degrees of freedom, *P p* value

Hemodynamic instability (transient or non-responding to fluid therapy) was present in 79.3% (1175/1482) of the total studied population. The studies with patients with established hemorrhagic shock [systolic blood pressure (SBP) below 90 mmHg] due to trauma 90.9% (791/870), rAAA 91.7% (355/387) and other 12.9% (29/225) were respectively described. Overall reported mortality within the group that was hemodynamically stabilized with REBOA was 49.2% (613/1246). Reported mortality among studies concerning trauma was 63.0% (545/865). Meta-analysis revealed a significant difference in mortality (*p* < 0.001) of REBOA compared with the mortality of patients treated with other means. It showed a risk difference of 0.27 (0.14–0.49) favoring REBOA (Fig. 2). In studies regarding rAAA mortality was 39.1% (61/156) and for miscellaneous causes 3.1% (7/225). There were no episodes of mortality reported among studies on post-partum hemorrhage, gastrointestinal bleeding, or with the use of balloon occlusion during pelvic and sacral surgery. Additional information can be found in supplemental Tables 1, 2 and 3.

### The effects of REBOA

**Fig. 3:**
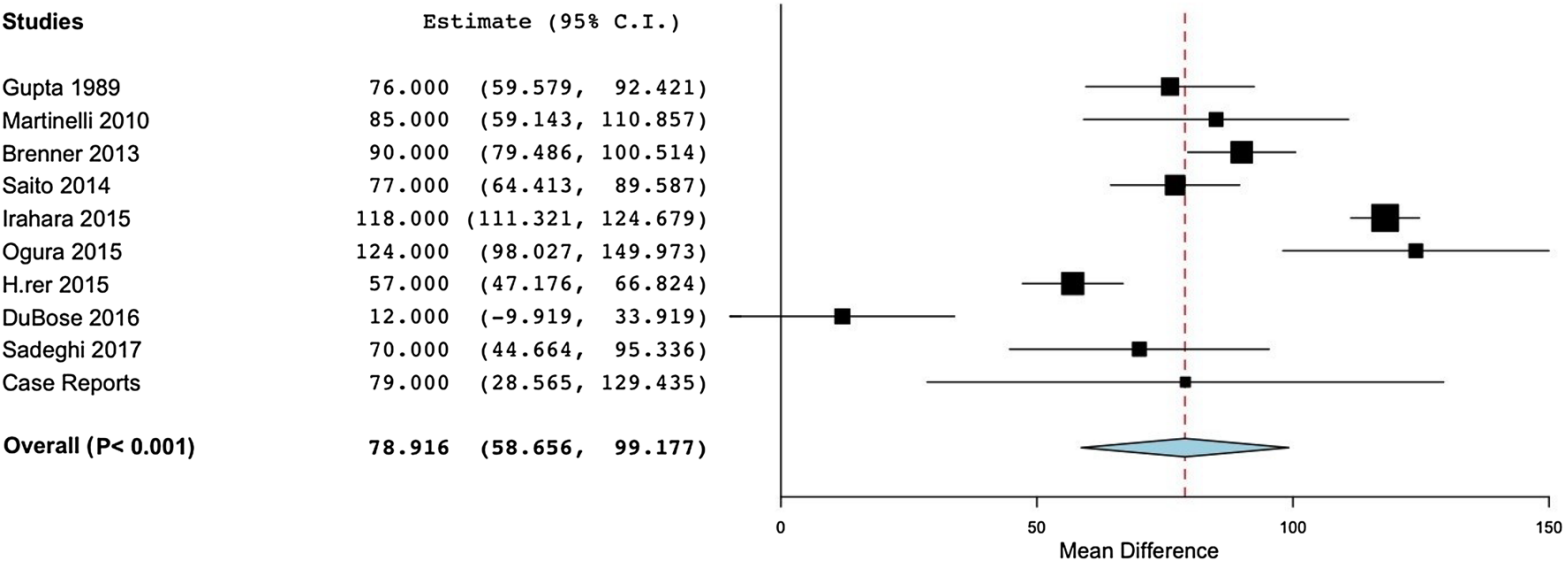
Meta-analysis of rise in SBP after REBOA use in trauma. *SBP* indicates systolic blood pressure in mmHg, *REBOA* resuscitative endovascular balloon occlusion of the aorta, *CI* confidence interval, *P p* value

The effect on SBP in hemodynamic instability, as seen in Table 2, was a significant increase of SBP by a mean of 78.9 mmHg in trauma (Fig. 3), 56.1 mmHg in rAAA and 52.4 mmHg other types of patients in established hemorrhagic shock, among the 23 studies [10, 12, 22, 25, 29–31, 34, 36, 40, 41, 44, 45, 47, 48, 52, 55, 58, 59, 62, 66, 71, 97, 104, 105, 111] reporting pre-REBOA and post-REBOA SBP values.

**Table 2:** Mortality, occlusion time and rise of systolic blood pressure with application of REBOA

	Number of studies	Total	Mortality	Unstable
Trauma	18	870	545/865 (63.0%)	791/870 (90.9%)
rAAA	50	387	61/156 (39.1%)	355/387 (91.7%)
Other	21	225	7/225 (3.1%)	29/225 (12.9%)
Total	89	1482	613/1246 (49.2%)	1175/1482 (79.3%)

	Art. insertion	Acces tech	occlusion time	Δ*P* mmHg
Trauma	Aortal 0	Percutaneous 310	11,953/230	Meta-analysis (*n* = 240)
	Axillary 0			
	Brachial 1	Cutdown 79	52.0 min	79.8 mmHg
	Carotid 1			
	Femoral 388			(pooled: 15,195/315)
rAAA	Aortal 7	Percutaneous 221	436/13	898/16
	Axillary 3			
	Brachial 2	Cutdown 99	33.5 min	56.1 mmHg
	Carotid 0			
	Femoral 312			
Other	Aortal 8	Percutaneous 165	10,319/159	681/13
	Axillary 0			
	Brachial 0	Cutdown 56	64.9 min	52.4 mmHg
	Carotid 0			
	Femoral 217			
Total (pooled)	Aortal 15	Percutaneous 696	22,708/432	16,773/344
	Axillary 3			
	Brachial 3	Cutdown 234	56.5 min	48.8 mmHg
	Carotid 1			
	Femoral 669			

	Art. insertion	Mean occlusion time		Number of studies
Trauma	Zone I	11,111/190 = 58.5 min		8
	Zone II	270/6 = 45.0 min		2
	Zone III	2175/32 = 68.0 min		8
rAAA	Zone I	90/3 = 30.0 min		2
	Zone II	30/1 = 30.0 min		2
	Zone III	31/2 = 15.5 min		2
Other	Zone I	205/4 = 51.3 min		4
	Zone II	0		0
	Zone III	10,114/189 = 53.5 min		10
Total	Zone I	11,496/197 = 58.4 min		15
	Zone II	305/7 = 43.6 min		3
	Zone III	12,320/223 = 55.2 min		20

*REBOA* indicates resuscitative endovascular balloon occlusion of the aorta, *rAAA* ruptured abdominal aneurysm, *Art.Ins* location of arterial incision, *tech* technique, *min* minutes, *ΔP* rise of systolic blood pressure in mmHg

REBOA balloon occlusion times were adequately reported in 35 studies [10, 12, 23, 25, 26, 29–34, 36, 38–41, 43–45, 47, 48, 55, 58, 62, 66, 68–71, 82, 83, 97, 101, 104, 105]. Occlusion times within the zones of occlusion were described in 15 studies examining Zone I occlusion times, where 196 patients had a median occlusion time of 58.4 min. Only three studies reported on occlusion in zone 2, with a mean of 43.6 min. The remaining nineteen studies examined 223 patients undergoing Zone III occlusion, with a median occlusion time of 55.2 min.

Of the studies included for review, 81 (92.0%) reported adequate data regarding arterial access and REBOA balloon deployment (Table 1). The femoral artery was the most commonly reported access site (96.8% of the patients) for REBOA, followed by direct aortic access (2.2% of the patients). Interestingly the largest study in our review, Norii et al (*n* = 452), did not report information on the access sites utilized [92]. Iatrogenic injuries related to REBOA use were 3.7% (32/870), 2.6% (10/387) and 5.3% (12/225) respectively on trauma, rAAA and miscellaneous causes (*p* = 0.21).

## Discussion

The current review is the largest systematic analysis of the literature on the subject of REBOA. A total of 89 studies, reporting on 1482 patients have been identified in the current published literature. The REBOA concept increases the SBP in hemorrhagic shock and is an adjunct for both endovascular and open repair in hemodynamic instability due to major exsanguination. The described iatrogenic injury rate related to REBOA is below 5%.

### Central traumatic major exsanguination

The most important question to answer is whether REBOA imparts a survival benefit in the in-hospital phase of care. Within the trauma literature, both non-survivors and survivors tended to have a higher burden of injury, presenting with gross physiologic derangements. The majority of studies lack a sound control group, with the exception of the studies by Norii et al. [92] and Moore et al. [88] In most studies, the study of Norii et al. [92] included, it was unclear whether REBOA was used in the context of a formal damage-control protocol, with consistent application, or whether it was used as a “last-ditch” attempt to salvage in hospital patients (e.g. ascending aortic ruptures) in civilian or military environment who were anticipated to have impending unfavorable outcome [14]. For this reason the study by Norii et al. [92], had to be excluded from the meta-analysis.

While our report suggests REBOA utilization has increased in recent years with results revealing a significant rise in blood pressure and a survival rate of approximately 40% with patients in shock (Table 2), the use of this adjunct is not universally embedded in hospital settings, not to mention the pioneering prehospital phase [15]. In the combat environment, major hemorrhage from central vascular injuries endures as the leading cause of death and most of the casualties decease before reaching a hospital [112–115]. REBOA can, therefore be an adjunct in the resuscitation of these major central/junctional hemorrhages in both the military as well as the civilian environment. Future research should focus on the usage of the REBOA concept in the prehospital phase, including selection and training of the right medical personnel for this important form of bleeding control.

### Ruptured acute aortic aneurysm

The treatment of choice for rAAA depends on hemodynamic, morphological characteristics as well as the availability of an endovascular team trained for EVAR procedures. Historically, patients in profound hemodynamic shock have been deemed not suitable for endovascular approach, because of the extra time involved in measuring and preparing for an endovascular procedure. The use of REBOA in these patients may extend the period needed to assemble the necessary staff for endovascular repair. It may even have potential application in the pre- or inter-hospital setting, to bridge the transport time to an endovascular center with sufficient expertise.

Three RCT’s have studied the optimal treatment for ruptured aneurysms, EVAR or open [116–120]. In the reported results of the ECAR and AJAX trials, EVAR was found to be equal to open surgical repair in terms of 30 day and 1-year mortality. In contrast, the IMPROVE trial concluded that a strategy of endovascular repair was not associated with significant reduction in either 30-day mortality or cost. The 2014 Cochrane review on this subject concluded that, based on available data at the time of analysis, there is no difference in the 30-day mortality outcomes between EVAR and open repair [121]. It is important to note, however, that none of the presently available trials have randomized high-risk patients with ruptured aneurysms, particularly those in shock or with low blood pressure [116]. These patients potentially represent the population most likely to benefit from early aortic balloon occlusion, possibly even in the pre-hospital phase or in the emergency department. This patient subset warrants further examination.

### Miscellaneous causes of major hemorrhage

Miscellaneous causes of significant hemorrhage, such as post-partum hemorrhage, gastro-intestinal bleeding or exsanguination during pelvic surgery, are also likely to benefit from early direct bleeding control to prevent the onset of the lethal triad (hypothermia, acidosis and coagulopathy). While the data on this subset of patients is limited, our observed mortality rate of lower than 5% among REBOA patients is suggestive that this technology has potentially important applications for these indications. Since shock was not presented in the studies regarding PPH, GI bleeding or pelvic surgery, additional examination of these patient subgroups is warranted.

### Limitations

While our review represents the largest of such effort in the medical literature, it does possess several limitations that must be addressed. The major limitation of the current review is the quality of the available evidence, which is limited. The risk of population bias in this systematic review is inevitable, to minimize best possible effects of heterogeneity and cohort overlap we provided a narrative description of prevalence and characteristics. A recent published review of Gamberini et al. [122] contained several papers that are not mentioned in our study [123–133], most of them based on the Japanese trauma registry. It was not possible to determine how much overlap there was with the studies we included from the Japanese registry. To prevent bias by double patient inclusion, these studies were not included in this analysis. The majority of the evidence identified consists of case reports and case series (Grade IV, V evidence) with only limited cohort studies and reviews identified (Grade II, III evidence). Most studies are at significant risk of bias, underestimating the true mortality and morbidity (including iatrogenic injuries). These findings should prompt efforts to improve on this by stimulating formal prospective evaluation.

REBOA is a potential lifesaving adjunct for resuscitation but prolonged occlusion of the aorta can lead to organ failure due to resulting ischemia–reperfusion injury. Consensus regarding safe occlusion times is not yet reached although animal studies indicate an optimal duration of 30 min. Occlusion time of 60 min or above is associated with an increased mortality [134, 135]. For prolonged field care or surgery, intermittent or partial occlusion could be a solution [71]. Three studies reported on occlusion times in zone 2 [40, 104, 111]. When the exact location of the bleeding is known, control in this zone could be needed in specific circumstances. The use of zone 2 as deployment zone is usually not recommended [136].

Prospective data collection is underway in the form of an American Association for the Surgery of Trauma sponsored observation study (AORTA), the European registry (ABOT), the Japanese registry (DIRECT-IABO) and the UK-REBOA Randomized Control Trial which should permit the consistent capture and reporting of REBOA-specific data such as indications, hemodynamic performance, outcome, cause of death, and morbidity [137–140].

Growing experience with the REBOA concept has proven its value in (acute) aortic repair, and expanding experience is gathering in the clinical implementation of this technology in major hemorrhage for trauma indications. Ongoing research should provide a much-needed higher quality of data in the coming years. These maturing experiences should yield improved data on the degree of hemorrhagic shock, specifics of the setting of use (pre- and in-hospital), the influence of use on organ failure, associated blood product requirements, encountered iatrogenic injuries and the specific techniques utilized for deployment (balloon occlusion time; full/partial /intermittent inflation approaches) [12, 104, 141].

## Conclusion

The REBOA concept has been used as an effective early hemorrhage control and resuscitation adjunct following traumatic abdominopelvic hemorrhage, hemorrhage arising from rAAA and significant bleeding from miscellaneous causes (post-partum, gastro-intestinal bleeding or exsanguination during pelvic surgery). Once placed in the correct aortic zone it has been shown to effectively raise the SBP in patients in major hemorrhagic shock. Despite these documented hemodynamic improvements, however, there remains a need for improved evidence supporting mortality benefit following REBOA use in the setting of traumatic hemorrhage. Presently available data, however, continues to suggest a potential value for REBOA use in austere environments or for patients in extremis that cannot be ignored. For this specific subset of patients, where the traditional alternative is likely death, the adage “do you need a RCT to prove that a parachute works” may prove worthy of further discussion. A feasibility study to setup a formal training program for obtaining vascular access and REBOA placement by non-vascular medical specialists has recently been conducted by this study group. This systematic review underscores that solid prospective evaluation of the REBOA concept remains a significant requirement for the establishment of optimal guidelines for REBOA employment in the management of hemorrhagic shock in any phase of medical care.

## Electronic supplementary material

Below is the link to the electronic supplementary material.

Supplementary material 1 (DOCX 71 KB)
