# Supplementary material for: A systematic review and meta-analysis of the use of resuscitative endovascular balloon occlusion of the aorta in the management of major exsanguination
Source: Eur J Trauma Emerg Surg. 2018 May 21;44(4):535–50. doi: 10.1007/s00068-018-0959-y (PMC6096615; doi:10.1007/s00068-018-0959-y)
Supplement: Supplementary file 1 — Supplementary material 1 (DOCX 71 KB) [file 68_2018_959_MOESM1_ESM.docx]

**Supplemental Table 1.** Details of studies concerning trauma patients

| **Reference** | **Year** | **Study type** | **N** | | **Aortic zone of placement** | | **Shock** | | **Mortality**  **(in-hosp)** | | **AO related iatrogenic injury** | | **Risk of bias** | | **OXLE** | **Art. Ins** | | **Acces tech**  **Perc/Cut** | | **Occl. Time in min**  **(total/pts)**  **type** | | | **Δ P mmHg**  **(total/pts)** |
| --- | --- | --- | --- | --- | --- | --- | --- | --- | --- | --- | --- | --- | --- | --- | --- | --- | --- | --- | --- | --- | --- | --- | --- |
| ***Case report (4)*** | |  |  | |  | |  | |  | |  | |  | |  |  | |  | |  | | |  |
| **Green** | 2014 | Trauma abdpel. hem | 1 | | III | | N | | Nil | | Nil | | NA | | V | Femoral | | 1/0 | | U  Coda (Cook) | | | U |
| **Matsuoka** | 2001 | Trauma abdpel. Hem | 1 | | I | | Y | | Nil | | Nil | | NA | | V | Carotid | | 1/0 | | 10  5fr Moiyan (Goodtech) | | | 10 |
| **Uchida** | 2014 | Trauma abdpel. hem | 1 | | III | | Y | | Nil | | Nil | | NA | | V | Femoral | | 1/0 | | 600 (intermittend)  IABO (Senko) | | | U |
| **Wolf** | 1986 | Trauma abdpel. hem | 1 | | III | | Y | | Nil | | Nil | | NA | | V | Brachial | | 0/1 | | 45  8/22 Fogarty (Edwards) | | | 110 |
| **TOTAL** |  |  | **4** | | **Ix1, IIIx3** | | **3/4** | | **0/4** | | **0** | |  | |  | **B1/C1/F2** | | **3/1** | | **218 (655/3)** | | | **60 (120/2)** |
|  |  |  |  | |  | |  | |  | |  | |  | |  |  | |  | |  | | |  |
| ***Case series (7)*** | |  |  | |  | |  | |  | |  | |  | |  |  | |  | |  | | |  |
| **Brenner** | 2013 | Trauma abdpel. hem | 6 | | Ix4, IIIx2 | | Y | | 2/6 | | Nil | | High | | IV | Femoral | | 3 / 3 | | 43 (258/6)  Coda (Cook) | | | 61  (367/6) |
| **Gupta** | 1989 | Trauma abdpel. Hem | 21 | | Ix21 | | Y | | 14/21 | | 1xfem a thrombosis | | High | | IV | Femoral | | 8/13 | | U  Percluder (Intervascular) | | | 51  (1,071/21) |
| **Hughes** | 1954 | Trauma abdpel. hem | 2 | | Ix2 | | Y | | 2/2 | | Nil | | High | | IV | Femoral | | 0/2 | | 25 (1pt)  10fr Dotter-Lukas | | | 52  (1pt) |
| **Irahara** | 2015 | Trauma abdpel. hem | 14 | | Ix14 | | Y | | 9/14 | | Nil | | High | | IV | Femoral | | 14/0 | | 161 (2,248/14)  Block balloon (Senko) | | | 58  (812/14) |
| **Martinelli** | 2010 | Trauma abdpel. hem | 13 | | IIIx13 | | Y | | 7/13 | | 1x fem a thrombosis, 1x balloon rupture | | High | | IV | Femoral | | 13/0 | | 70 (915/13)  20mm Berstein (Boston) | | | 70  (910/13) |
| **Ogura** | 2015 | Trauma abdpel. hem | 35 | | Ix35 | | Y | | 16/35 | | Nil | | High | | IV | Femoral | | 7/0 | | 80 (560/7)  U | | | 52  (364/7) |
| **Wang** | 2013 | Trauma abdpel. hem | 5 | | IIIx5 | | Y | | U | | Nil | | High | | IV | Femoral | | 0/5 | | 51 (255/5)  Fogarty | | | U |
| **TOTAL** |  |  | **96** | | **Ix76, IIIx20** | | **96/96** | | **50/91** | | **3** | |  | |  | **F68** | | **45/23** | | **93 (4,261/46)** | | | **45 (2,764/62)** |
|  |  |  |  | |  | |  | |  | |  | |  | |  |  | |  | |  | | |  |
| ***Cohort study (7) (Low 2^nd^ x)*** | | | |  | |  | |  | |  | |  | |  | | |  | |  | |  |  | |
| **DuBose** | 2016 | Trauma | 46 | | Ix33, IIx1, IIIx8 | | 21/46 | | 13/46 | | 2x embolism, 1xpseudo aneurysm | | High | | IV | Femoral | | 23/23 | | 20 (920/46)  Miscellaneous | | | 67  (3082/46) |
| **Hörer** | 2015 | Trauma | 7 | | NA | | Y | | 2/7 | | 3x (2x perf. (iliaca/fem), uncontroled ECMO removal, | | High | | IV | Femoral | | 6/1 | | 28 (196/7)  Coda (Cook) | | | 40  (280/7) |
| **Hörer** | 2017 | Trauma | 96 | | Ix86, IIx3, IIIx3 | | 43/65 | | 54/96 | | 13 (miscellaneous) | | High | | IV | Femoral | | 90/6 | | U  Miscellaneous | | | 40  (3,840/96) |
| **Low** | 1986 | Trauma Abd-pelvic | 15 | | Ix15 | | Y | | 13/15 | | 3x perc access fail, 1x cut down failure | | High | | IV | Femoral | | 4/11 | | 50 (150/3)  Percluder (Intervascular) | | | 55  (110/2) |
| **Matsumara** | 2017 | Trauma | 106 | | Ix99, IIx5, IIIx2 | | Y | | 38/106 | | Nil | | Low | | III | Femoral | | 104/1 | | 50 (5,300/106)  Miscellaneous | | | 52 (3,918/76) |
| **Moore** | 2015 | Trauma abdpel. hem | 24 | | Ix19, IIIx5 | | Y | | 15/24 | | Nil | | High | | IV | Femoral | | 12/12 | | U | | | U |
| **Norii** | 2015 | Trauma abdpel. hem | 452 | | U | | Y | | 343/452 | | Nil | | High | | III | U | | U | | U | | | U |
| **Saito** | 2014 | Blunt trauma | 24 | | U | | Y | | 17/24 | | 5x failure of defl/1x iliac injury | | High | | IV | Femoral | | 23/1 | | 25 (469/19)  IAOB (Mera) | | | 45  (1,080/24) |
| **TOTAL** |  |  | **770** | | **Ix252, IIx9, IIIx18** | | **692/770** | | **495/770** | | **29** | |  | |  | **F318** | | **262/55** | | **39 (7,037/181)** | | | **49 (12,310/251)** |
| **GRAND TOTAL**  **(n=18 studies)** | |  | **870** | | **Ix329, IIx9, IIIx41** | | **791/870**  **(90.9%)** | | **545/865**  **(63.0%)** | | **32** | |  | |  | **B1/C1/F388** | | **310/79** | | **52.0 min**  **(11,953/230)** | | | **48.2 mmHg (15,194/315)** |

*^EVAR^* ^indicates endovascular aneurysm repair,^ *^N^*^: number,^ *^AO^*^: “Arbeitsgemeinschaft für Osteosynthesefragen”,^ *^OXLE^*^: Oxford level of evidence,^ *^Art.Ins^*^: location of arterial incision,^ *^tech^*^: technique,^ *^Perc^*^: percutaneous,^ *^Occl^*^: occlusion,^ *^SD^*^: standard deviation,^ *^Δ P mmHg^*^: pressure difference in mmHg,^ *^rAAA^*^: ruptured abdominal aneurysm,^ *^abdpel hem^*^: abdominal/pelvic haemorrhage,^ *^PPH^*^: post partum haemorrhage,^ *^(U)GI^*^: (upper) gasto intestinal,^ *^OR^*^: operation room,^ *^r.^*^: ruptured,^ *^iatr/gyn^*^: iatrogenic/gynaecological,^ *^Y^*^: yes,^ *^NA^*^: not applicable,^ *^U^*^: unknown,^ *^nm^*^: not measurable,^ *^Ao:^* ^aortal,^ *^Ax^*^: axillary,^ *^B^*^: brachial,^ *^C^*^: carotis,^ *^F^*^: femora,^ *^(I)AOB^*^: (intra) aortic occlusion balloon,^ *^bal^*^: balloon,^ *^cath^*^: catheter,^ *^pts^*^: patients,^ *^fem a^*^.: femoral artery,^ *^def^*^: deflation,^ *^pt(s)^*^: patient(s).^

**Supplemental Table 2.** Details of studies concerning rAAA patients

| **Reference** | **Year** | **Study type** | **N** | **Aortic zone of placement** | **Shock** | **Mortality (in-hosp)** | **AO related iatrogenic injury** | **Risk of bias** | **OXLE** | **Art. Ins** | **Acces tech**  **Perc/Cut** | **Occl. Time in min**  **(total/pts)**  **type** | **Δ P mmHg**  **(total/pts)** |
| --- | --- | --- | --- | --- | --- | --- | --- | --- | --- | --- | --- | --- | --- |
| ***Case Report (10)*** | |  |  |  |  |  |  |  |  |  |  |  |  |
| **Armour** | 1978 | rAAA | 1 | I | Y | 0 | Nil | NA | V | Femoral | 0/1 | U | U |
| **Cakir** | 2014 | rAAA | 1 | I | Y | 0 | Nil | NA | V | Femoral | 1/0 | U  34mm Amplatzer (St. Jude) | 80 |
| **Heimbecker** | 1964 | rAAA | 1 | III | Y | 0 | Nil | NA | V | Brachial | 0/1 | U  U | U |
| **Hesse** | 1962 | rAAA | 1 | III | Y | 1/1 | Nil | NA | V | Aortal | 0/1 | 12  20F Foley Cath. Balloon | U  n |
| **Howard** | 1972 | rAAA | 1 | III | Y | 0 | Nil | NA | V | Aortal | 0/1 | U  30F Foley Cath. Balloon | U |
| **Lai** | 2008 | rAAA | 1 | I | Y | 0 | Nil | NA | V | Femoral | 0/1 | 50  Coda (Cook) | 60 |
| **Malina** | 2005 | rAAA | 1 | I | Y | 0 | Nil | NA | V | Femoral | 1/0 | 30  U | U |
| **Namura** | 2001 | rAAA | 1 | III | Y | 0 | Nil | NA | V | Brachial | 0/1 | 19  U | 20 |
| **Schumacher** | 2004 | rAAA | 1 | II | Y | 0 | Nil | NA | V | Femoral | 0/1 | 35  46mm Reliant (Medronic) | 5 |
| **Smith** | 1972 | rAAA | 1 | III | Y | 0 | Nil | NA | V | Axillary | 0/1 | U  8/22 Fogarty (Edwards) | U |
| **TOTAL** |  |  | **10** | **Ix5, IIx1, IIIx5** | **10/10** | **1/10** | **0** |  |  | **Ao2/Ax1/B2/F5** | **2/8** | **29 (146/5)** | **41 (165/4)** |
|  |  |  |  |  |  |  |  |  |  |  |  |  |  |
| ***Case series (14)*** | |  |  |  |  |  |  |  |  |  |  |  |  |
| **Arthurs** | 2006 | rAAA | 3 | Ix3 | N | 0/3 | Nil | High | IV | Femoral | 3/0 | 10 (1pt)  33mm AOB (U) | 40  (1pt) |
| **Delalieux** | 2010 | rAAA | 1 | U | Y | U | Nil | High | IV | Femoral | U | U  Reliant (Medtronic) | U |
| **Greenberg** | 2000 | rAAA | 2 | Ix2 | Y | 0/2 | Nil | High | IV | Axillary | 0/2 | U | U |
| **Guo** | 2009 | rAAA | 4 | U | Y | U | Nil | High | IV | U | U | U | U |
| **Hinchliffe** | 2001 | rAAA | 2 | Ix2 | Y | U | Nil | High | IV | Femoral | 2/0 | U  30mm Valvuloplasty Bal. | U |
| **Lagana** | 2006 | rAAA | 3 | IIx3 | Y | U | Nil | High | IV | Femoral | 0/3 | U  Equalizer (Boston Scientific) | U |
| **Lee** | 2008 | rAAA | 3 | Unk | Y | U | Nil | High | IV | Brachial | 0/3 | U | U |
| **Matsuda** | 2003 | rAAA | 11 | III | Y | 3/11 | 3x bal. rupture (2ns), 2x embolic complication | High | IV | Brachial | 0/11 | U  8/22 Fogarty (Edwards) | 63 (693/11) |
| **Mayer** | 2009 | rAAA | 19 | Ix19 | Y | U | 1x embolic complication | High | IV | Femoral | 19/0 | U  46mm Reliant (Medtronic) | U |
| **Ng** | 1977 | rAAA | 5 | IIIx5 | Y | 3/5 | Nil | High | IV | Brachial | 0/5 | U  8/22 Fogarty (Edwards) | U |
| **Ohki** | 2000 | rAAA | 9 | Ix9 | Y | U | Nil | High | IV | Brachial | 0/9 | U  40mm Aortic (Meditech) | U |
| **Philipsen** | 2009 | rAAA | 12 | Ix12 | Y | 1/12 | Nil | High | IV | Femoral | 12/0 | U  46mm Reliant (Medtronic) | U |
| **Sensenig** | 1981 | rAAA | 3 | Ix2,/IIx1 | Y | 0/3 | Nil | Low | IV | F2/Ao1 | 0/3 | U  8/22 Fogarty (Edwards) | U |
| **Taheri** | 1988 | rAAA | 2 | IIIx2 | N/Y | 0/2 | Nil | Low | IV | Aortal | 0/2 | U  30cc Foley Cath. | U |
| **TOTAL** |  |  | **79** | **Ix49, IIx4, IIIx8** | **75/79** | **7/38** | **6** |  |  | **Ao3/Ax2/B28/F42** | **36/38** | **10 (1pt)** | **61 (733/12)** |
|  |  |  |  |  |  |  |  |  |  |  |  |  |  |
| ***Cohort study (26)*** | |  |  |  |  |  |  |  |  |  |  |  |  |
| **Alsac** | 2005 | rAAA  EVAR vs Open | 1 | Ix1 | Y | U | Nil | High | IV | Femoral | 0/1 | U  Reliant (Medtronic) | U |
| **Anain** | 2007 | rAAA | 12 | Ix12 | Y | 5/12 | Nil | High | IV | Femoral | 0/11 | U  Reliant or Coda | U |
| **Carafiello** | 2012 | rAAA | 4 | U | Y | U | Nil | High | IV | Femoral | 0/4 | U  AOB (Boston Scientific) | U |
| **Coppi** | 2006 | rAAA | 4 | IIx4 | Y | U | Nil | High | IV | Femoral | 0/4 | U  Prototype (Edwards) | U |
| **Dalainas** | 2006 | rAAA | 28 | IIx28 | 5/28 | 13/28 | Nil | High | IV | B2, F26 | 28/0 | U  27,33 or 40mm AOB | U |
| **Djavani G.** | 2011 | rAAA | 2 | U | Y | 1/2 | Nil | High | IV | U | U | U | U |
| **Gerassimidis** | 2008 | rAAA | 2 | U | Y | U | Nil | High | IV | Femoral | 0/2 | U  Reliant | U |
| **Holst** | 2009 | rAAA | 23 | Ix23 | Y | U | 2x SMA coverage | High | IV | Femoral | 23/0 | U  Coda or large diam. latex | U |
| **Ioannidis** | 2012 | rAAA | 1 | U | Y | U | Nil | High | IV | U | U | U | U |
| **Karkos** | 2008 | rAAA | 2 | U | Y | U | Nil | High | IV | Femoral | 2/0 | U | U |
| **Larzon** | 2005 | rAAA | 13 | Ix13 | Y | U | Nil | High | IV | Ao2/F11 | 11/2 | 40 (280/7)  Equalizer Balloon Catheter, (Boston Scientific) | U |
| **Low** | 1986 | rAAA | 5 | Ix5 | Y | 1/5 | 1x cannulation failure. 1x coiling of cath | High | IV | Femoral | 12/11 | U  Percluder | U |
| **Mayer** | 2012 | rAAA | 62 | Ix62 | Y | U | Nil | High | III | Femoral | 62/0 | U  Pruitt occlusion catheter (LeMaitre Vascular) | U |
| **Mehta 2005** | 2005 | rAAA | 7 | Ix7 | Y | U | Nil | High | IV | U | U | U | U |
| **Mehta 2013** | 2013 | rAAA | 23 | Ix23 | Y | U | Nil | High | IV | Femoral | 23/0 | U | U |
| **Moore** | 2006 | rAAA | 7 | IIx7 | Y | 1/7 | Nil | High | IV | Femoral | 0/7 | U  Reliant or Coda | U |
| **Mukherjee** | 2014 | rAAA | 3 | Ix3 | Y | U |  | High | IV | Femoral | U | U | U |
|  | 2014 | rAAA (hybrid group) | 8 | Ix8 | 3/8 | 0/8 | Nil | Low | IV | Femoral | U | U | U |
| **Nedeau** | 2012 | rAAA | 11 | U | Y | U | Nil | High | IV | Femoral | 11/0 | U | U |
| **Ockert** | 2007 | rAAA | 2 | U | Y | U | Nil | High | IV | Femoral | 0/2 | U | U |
| **Peppelenbosch** | 2005 | rAAA | 7 | II | Y | U | Nil | High | IV | Femoral | U | U | U |
| **Raux** | 2015 | rAAA | 32 | Ix32 | Y | 22/32 | Nil | Low | III | Femoral | U | U  46mm Reliant | U |
| **Resch** | 2003 | rAAA | 5 | U | Y | U | Nil | High | IV | Femoral | 0/5 | U | U |
| **Sarac** | 2011 | rAAA | 3 | U | Y | 0/3 | Nil | High | IV | Femoral | 0/4 | U | U |
| **Starnes** | 2010 | rAAA | 11 | U | Y | 7/11 | Nil | High | IV | Femoral | 11/0 | U  Coda (Cook Medical) | U |
| **Veith** | 2002 | rAAA | 10 | U | Y | U | Nil | High | IV | U | U | U | U |
| **Veith** | 2003 | rAAA | 10 | Ix10 | Y | U | Nil | High | IV | U | U | U  Compliant with 14fr sheat  (Meditech) | U |
| **TOTAL** |  |  | **298** | **Ix200, IIx38, IIIx0** | **270/298** | **53/108** | **4** |  |  | **Ao2/B2/F265** | **183/53** | **40 (280/7)** | **U** |
| **GRAND TOTAL**  **(n=50 studies)** | |  | **396** | **Ix252, IIx46, IIIx13** | **355/387** | **61/156**  **(39.1%)** | **10** |  |  | **Ao7/Ax3/ B32/F312** | **221/99** | **33.5 min (436/13)** | **56.1 mmHg (898/16)** |

*^EVAR^* ^indicates endovascular aneurysm repair,^ *^N^*^: number,^ *^AO^*^: “Arbeitsgemeinschaft für Osteosynthesefragen”,^ *^OXLE^*^: Oxford level of evidence,^ *^Art.Ins^*^: location of arterial incision,^ *^tech^*^: technique,^ *^Perc^*^: percutaneous,^ *^Occl^*^: occlusion,^ *^SD^*^: standard deviation,^ *^Δ P mmHg^*^: pressure difference in mmHg,^ *^rAAA^*^: ruptured abdominal aneurysm,^ *^abdpel hem^*^: abdominal/pelvic haemorrhage,^ *^PPH^*^: post partum haemorrhage,^ *^(U)GI^*^: (upper) gasto intestinal,^ *^OR^*^: operation room,^ *^r.^*^: ruptured,^ *^iatr/gyn^*^: iatrogenic/gynaecological,^ *^Y^*^: yes,^ *^NA^*^: not applicable,^ *^U^*^: unknown,^ *^nm^*^: not measurable,^ *^Ao:^* ^aortal,^ *^Ax^*^: axillary,^ *^B^*^: brachial,^ *^C^*^: carotis,^ *^F^*^: femora,^ *^(I)AOB^*^: (intra) aortic occlusion balloon,^ *^bal^*^: balloon,^ *^cath^*^: catheter,^ *^pts^*^: patients,^ *^fem a^*^.: femoral artery,^ *^def^*^: deflation,^ *^pt(s)^*^: patient(s).^

**Supplemental Table 3.** Details of studies concerning other patients

| **Reference** | **Year** | **Study type** | **N** | **Aortic zone of placement** | **Shock** | **Mortality (in-hosp)** | **AO related iatrogenic injury** | **Risk of bias** | **OXLE** | **Art. Ins** | **Acces tech**  **Perc/Cut** | **Occl. Time in min (SD)**  **type** | **Δ P mmHg (SD)[95%]** |
| --- | --- | --- | --- | --- | --- | --- | --- | --- | --- | --- | --- | --- | --- |
| ***Case Report (14)*** |  |  |  |  |  |  |  |  |  |  |  |  |  |
| **Bell-Thomas** | 2003 | PPH | 1 | III | Y | Nil | Nil | NA | V | Femoral | 0/1 | U  10fr Aortic (BVM Medical) | U |
| **D’Hondt** | 2008 | r. aneurysma aortobifem graft | 1 | III | Y | Nil | Nil | NA | V | Femoral | 1/0 | 5  20mm AOB | U |
| **Harma** | 2004 | PPH | 1 | III | Y | Nil | Nil | NA | V | Femoral | 1/0 | 6  IABP (Datascope) | 100 |
| **Hill** | 2010 | UGI bleeding | 1 | I | Y | Nil | Nil | NA | V | Femoral | 1/0 | U  14mm Angioplasty (Bard) | U |
| **Karkos** | 2001 | UGI bleeding | 1 | I | Y | Nil | Nil | NA | V | Femoral | 0/1 | 30  40cc IABP | 80 |
| **Lee** | 2016 | GI bleeding | 1 | I | Y | Nil | Nil | NA | V | Femoral | 1/0 | 15  REBOA | 51 |
| **Masamoto** | 2009 | PPH | 1 | III | N | Nil | Nil | NA | V | Femoral | 1/0 | 80  30mm Aortic (Forte) | U |
| **Menke** | 2010 | r-Para-anastomotic iliac aneur. | 1 | III | Y | Nil | Nil | NA | V | Femoral | U | U  46mm Reliant (Medtronic) | U |
| **Ozgiray** | 2009 | Pelvic bleeding peri-OR | 1 | III | N | Nil | Nil | NA | V | Femoral | 1/0 | U  Balloon Dilation Cath. | U |
| **Paull** | 1995 | PPH | 1 | III | N | Nil | Nil | NA | V | Femoral | 1/0 | 15  18mm on 8.5fr Aortic (Cook) | U |
| **Pettersson** | 2003 | r. pseudo aneurysma aorta asc | 1 | I | Y | Nil | Nil | NA | V | Femoral | 0/1 | 10  AOB | U |
| **Shigesato** | 2015 | UGI | 1 | I | Y | Nil | Nil | NA | V | Femoral | 1/0 | 150  8fr Fogarty IAOB (Edwards) | 90 |
| **Soda** | 2010 | Pelvic bleeding peri-OR | 1 | III | N | Nil | Nil | NA | V | Femoral | U | 15  Aortic balloon cath. | U |
| **Xiong** | 2014 | Pelvic bleeding peri-OR | 1 | III | N | Nil | Nil | NA | V | Femoral | 1/0 | U  AOB | U |
| **TOTAL** |  |  | **14** | **Ix5, IIIx9** | **9/14** | **0/14** | **0** |  |  | F14 | **9/3** | **36 (326/9)** | **80 (321/4)** |
|  |  |  |  |  |  |  |  |  |  |  |  |  |  |
| ***Case series (4)*** |  |  |  |  |  |  |  |  |  |  |  |  |  |
| **Sovik** | 2012 | PPH | 6 | IIIx6 | Y | Nil | 1x Aortic injury | High | IV | Femoral | 6/0 | 27.3 (109/4)  30mm Aortic (NuMed) | 36.6  220/6 |
| **Xue-Song** | 2010 | Pelvic bleeding peri-OK | 9 | IIIx9 | N | Nil | Nil | High | IV | Femoral | 9/0 | 50  Balloon Dilation Cath. | U |
| **Yang** | 2008 | Pelvic bleeding peri-OR | 12 | IIIx12 | N | Nil | Nil | High | IV | Femoral | 12/0 | 60 /85 median  Sizing (AGA Medical) | U |
| **Zhang** | 2007 | Pelvic bleeding peri-OR | 5 | IIIx5 | N | Nil | Nil | High | IV | Femoral | 5/0 | 51 (255/5)  Sizing (NuMed) | 60  (1 pt) |
| **TOTAL** |  |  | **32** | **IIIx32** | **6/32** | **0/32** | **1** |  |  | F32 | **32/0** | **51 (1,534/30)** | **40 (280/7)** |

| ***Cohort study (3) (Low 3^rd^x/Hörer 2^nd^x)*** | | |  |  |  |  |  |  |  | |  |  |  |  |  |
| --- | --- | --- | --- | --- | --- | --- | --- | --- | --- | --- | --- | --- | --- | --- | --- |
| **Hörer** | 2015 | Iatr/gyn | 4 | NA | Y | 2/4 | Nil | High | IV | | Femoral | 4/0 | U  33-mm Balloon (Equalizer) or  Coda (Cook) | 40  (80/2) |  |
| **Low** | 1986 | Other | 2 | Ix2 | Y | 2/2 | Nil | High | IV | | Femoral | U | U  Percluder (Intervascular) | U |  |
| **Luo** | 2013 | Pelvic bleeding peri-OR | 45 | IIIx45 | N | 0/45 | 3x fem a thrombosis | High | IV | | Femoral | 0/45 | U | U |  |
| **Takagi** | 2003 | Aortic Arch repair  (8/30 ruptured) | 8 | Ix8 | Y | 3/8 | Nil | High | | IV | Aortic Arch | 0/8 | U  AABOC MD21010  (Sumitomo Bakelite) | U | |
| **Tang** | 2010 | Pelvic bleeding peri-OR | 120 | IIIx120 | N | 0/120 | 3x fem a embolism, 5x puncture site hematoma | High | III | | Femoral | 120/0 | 70.5 (8459/120)  Maxi LD (Cordis) | U |  |
| **TOTAL** |  |  | **179** | **Ix10, IIIx165** | **14/179** | **7/179**  **(3.9%)** | **11** |  |  | | **Ao8/F171** | **124/53** | **70 (8,459/120)** | **40 (80/2)** |  |
| **GRAND TOTAL**  **(n=21 studies)** | |  | **225** | **Ix15, IIx0, IIIx206** | **29/225** | **7/225**  **(3.1%)** | **12** |  |  | | **Ao8 / F217** | **165/56** | **64,9 min**  **(10,319/159)** | **52.4mmHg (681/13)** |  |

*^EVAR^* ^indicates endovascular aneurysm repair,^ *^N^*^: number,^ *^AO^*^: “Arbeitsgemeinschaft für Osteosynthesefragen”,^ *^OXLE^*^: Oxford level of evidence,^ *^Art.Ins^*^: location of arterial incision,^ *^tech^*^: technique,^ *^Perc^*^: percutaneous,^ *^Occl^*^: occlusion,^ *^SD^*^: standard deviation,^ *^Δ P mmHg^*^: pressure difference in mmHg,^ *^rAAA^*^: ruptured abdominal aneurysm,^ *^abdpel hem^*^: abdominal/pelvic haemorrhage,^ *^PPH^*^: post partum haemorrhage,^ *^(U)GI^*^: (upper) gasto intestinal,^ *^OR^*^: operation room,^ *^r.^*^: ruptured,^ *^iatr/gyn^*^: iatrogenic/gynaecological,^ *^Y^*^: yes,^ *^NA^*^: not applicable,^ *^U^*^: unknown,^ *^nm^*^: not measurable,^ *^Ao:^* ^aortal,^ *^Ax^*^: axillary,^ *^B^*^: brachial,^ *^C^*^: carotis,^ *^F^*^: femora,^ *^(I)AOB^*^: (intra) aortic occlusion balloon,^ *^bal^*^: balloon,^ *^cath^*^: catheter,^ *^pts^*^: patients,^ *^fem a^*^.: femoral artery,^ *^def^*^: deflation,^ *^pt(s)^*^: patient(s).^
